# Serum vitamin D levels are decreased and associated with thyroid volume in female patients with newly onset Graves’ disease

**DOI:** 10.1007/s12020-012-9679-y

**Published:** 2012-05-01

**Authors:** Tetsuyuki Yasuda, Yasuyuki Okamoto, Noboru Hamada, Kazuyuki Miyashita, Mitsuyoshi Takahara, Fumie Sakamoto, Takeshi Miyatsuka, Tetsuhiro Kitamura, Naoto Katakami, Dan Kawamori, Michio Otsuki, Taka-aki Matsuoka, Hideaki Kaneto, Iichiro Shimomura

**Affiliations:** 1 Department of Metabolic Medicine, Osaka University Graduate School of Medicine, 2-2 Yamadaoka, Suita, Osaka, 565-0871 Japan; 2 Sumire Clinic, Osaka, Japan

## Introduction

It has been shown that vitamin D deficiency is associated with autoimmune diseases, including rheumatoid arthritis (RA), systemic lupus erythematosus (SLE), inflammatory bowel disease (IBD), multiple sclerosis (MS) and type 1 diabetes (T1DM), and that vitamin D supplementation prevents the onset and/or development of these autoimmune diseases [1]. Furthermore, it was reported more recently that patients with Hashimoto’s thyroiditis, an autoimmune thyroid disease had lower vitamin D levels [2]. However, there are few studies examining vitamin D status in patients with newly onset Graves’ disease. In the present study, we evaluated the vitamin D status in female patients with newly onset GD and the association of serum vitamin D levels with the clinical factors related to GD.

## Patients and methods

A total of 26 female patients with newly onset GD were consecutively recruited from the subjects who attended the Sumire clinic to check the thyroid disease in winter and spring periods in 2011. GD was diagnosed by clinical and biochemical symptoms of hyperthyroidism, the presence of diffused goiter and TRAb positivity. Forty-six control healthy female subjects who had normal thyroid function without antithyroid peroxidase antibody (TPOAb) and antithyroglobulin antibody (TgAb) were recruited in the same period. This study was approved by the ethical committee of Sumire clinic, and informed consent was obtained from all patients. Vitamin D status was evaluated by the measurement of serum 25(OH)D_3_ levels by the competitive protein binding assay (Mitsubishi chemical medience, Tokyo, Japan). Vitamin D deficiency in our study was defined as levels of 25(OH)D_3_ below 15 ng/ml, as previously described [1]. Serum free T3 (FT3) and free T4 (FT4) levels were measured by competitive enzyme immunoassay (Tosoh, Tokyo, Japan). Serum TSH, TRAb, TPOAb, and TgAb levels were measured by two-site immunoenzymometric assay (Tosoh, Tokyo, Japan). Thyroid volume was measured by ultrasonography. Statistical analyses were performed by Stat-View statistical software (Version. 5.0 for Windows; Abacus Concepts, Berkeley, CA). A two-sided value of *P* < 0.05 was considered statistically significant.

## Results

Clinical characteristics in GD patients and control subjects were shown in Table 1. All serum TSH levels, and FT3 and FT4 levels in GD patients were below 0.01 μIU/ml and above upper limit of normal (3.8 pg/ml and 1.63 ng/dl), respectively. Serum TRAb levels in GD patients were 19.9 ± 18.1 IU/l. On the other hand, all of serum TSH, FT3, and FT4 levels in control subjects were within normal range (1.71 ± 0.96 μIU/ml, 2.76 ± 0.33 pg/ml, and 1.20 ± 0.17 ng/dl, respectively).

**Table 1:** Clinical characteristics

Number of subjects	GD patients	Control subjects	*P*
	26	46	
Age (years)	37.3 ± 13.0	44.3 ± 18.1	n.s.
BMI (kg/m^2^)	21.3 ± 4.0	22.2 ± 4.8	n.s.
Thyroid volume (cm^3^)	36.5 ± 19.4	15.5 ± 8.2	<0.0001
Ca (mg/dl)	9.9 ± 0.4	9.9 ± 0.5	n.s.
Intact-PTH (pg/ml)	31.8 ± 19.3	32.4 ± 14.1	n.s.
25(OH)D_3_ (ng/ml)	14.4 ± 4.9	17.1 ± 4.1	<0.05

Data are means ± SD

To examine an involvement of vitamin D in the pathogenesis of GD, we evaluated the vitamin D status in female patients with newly onset GD and the association of serum vitamin D levels with various GD-related clinical factors. As shown in Table 1, serum 25(OH)D_3_ levels were significantly lower in GD patients compared to control subjects (14.4 ± 4.9 vs. 17.1 ± 4.1 ng/ml, *P* < 0.05). The prevalence of vitamin D deficiency in GD patients was significantly higher compared to control subjects (65.4 vs. 32.4%, *P* < 0.05). Serum 25(OH)D_3_ levels were significantly associated with serum calcium levels (*r* = 0.49, *P* < 0.05) and intact parathyroid hormone levels (*r* = −0.50, *P* < 0.05) in GD patients. In addition, there was significant association between serum 25(OH)D_3_ levels and thyroid volume (*r* = −0.45, *P* < 0.05), but not thyroid function and TRAb levels in GD patients. Although further study would be necessary to conclude, these results suggest that the vitamin D status may be involved in the pathogenesis of GD.

## Discussion

To our best knowledge, this is the first report showing the decreased vitamin D levels in newly onset GD patients and the significant association between vitamin D status and thyroid volume, a clinical factor related to the pathogenesis of GD. Because it has been reported that vitamin D status is not changed by the treatment for GD [3], it is unlikely that decreased vitamin D status in patients with newly onset GD is induced by hyperthyroidism.

Vitamin D is known for its primary role in bone and mineral homeostasis, and it has been shown recently that its deficiency is associated with various diseases such as cardiovascular disease, cancer, infection, and adiposity as well as osteoporosis [4]. Interestingly, it has been shown recently that vitamin D has potent immunomodulatory effects and plays important roles in the pathogenesis of autoimmune diseases. Vitamin D inhibits the production of Th1 polarizing cytokine (IL-12), thereby indirectly shifting the polarization of T cells from a Th1 toward a Th2 phenotype. In the CD4^+^ T cell response, vitamin D directly inhibits the production of Th1 cytokines (IL2 and IFN-γ), and enhances Th2 cytokine (IL-4) production [1]. In addition, recent numerous studies have shown the relation of vitamin D and various autoimmune diseases. Vitamin D receptor (VDR) gene polymorphisms and vitamin D status are associated with different autoimmune diseases such as T1DM, MS, and IBD [5–7]. Furthermore, vitamin D supplementation prevented the onset and/or development of several kinds of autoimmune diseases in humans and animal models [1]. These results suggested that vitamin D deficiency might cause the onset and/or development of several kinds of autoimmune diseases.

GD is an autoimmune thyroid disease in which TSH receptor autoantibodies cause hyperthyroidism. The pathogenesis of GD is complicated and involved in multiple genetic and environmental factors. It has been thought that GD occurs with the infiltration of T cells in the thyroid gland as a result of these factors. Subsequently, T cells elaborate various cytokines leading to the production of TSH receptor autoantibodies. Especially, helper T cells produce various cytokines, including IFN-γ which induces thyrocytes to express major histocompatibility complex class II surface HLA-DR antigens and renders them susceptible to immunologic attack. Although HLA-DR antigens are not normally expressed on thyroid cells, in GD, the thyroid follicular cells present HLA-DR antigens on their surface with potential involvement in triggering autoimmune process [2]. Recent studies have demonstrated a role of vitamin D in GD. (i) Vitamin D related gene polymorphisms such as VDR gene and vitamin D binding protein gene are associated with GD. (ii) Vitamin D deficiency modulates Graves’ hyperthyroidism induced by thyrotropin receptor immunization in BALB/c mice. (iii) Vitamin D analog inhibits inflammatory responses in human thyroid cells and T cells [8–12]. Although further study would be necessary to conclude, these and our present results suggest that decreased vitamin D levels may cause the onset and/or development of GD as well as several kinds of autoimmune diseases.

In conclusion, vitamin D levels in female patients with newly onset GD are decreased and significantly associated with thyroid volume. It is noted, however, this study is cross-sectional survey with a small number of subjects, and limited in its ability to conclude that vitamin D status is directly related to the pathogenesis of GD. Therefore, the direct role of vitamin D in patients with GD should be examined by further prospective clinical studies by the treatment of vitamin D and experimental studies.
